# The Prevalence of Vitamin D Deficiency among Cancer Survivors in a Nationwide Survey of the Korean Population

**DOI:** 10.1371/journal.pone.0129901

**Published:** 2015-06-05

**Authors:** Myueng Guen Oh, Mi Ah Han, Jong Park, So Yeon Ryu, Seong-Woo Choi

**Affiliations:** 1 Department of Internal Medicine, Jeongup Asan Hospital, Jeongup, Korea; 2 Department of Preventive Medicine, College of Medicine, Chosun University, Gwangju, Korea; University of Alabama at Birmingham, UNITED STATES

## Abstract

**Background:**

Recent studies have shown that inadequate vitamin D levels are associated with a poor cancer prognosis, but data regarding actual vitamin D levels in cancer survivors are limited. This study investigated the vitamin D levels and prevalence of vitamin D deficiency among Korean cancer survivors compared with non-cancer controls, and identified the factors associated with vitamin D deficiency.

**Methods:**

Using the Korea National Health and Nutrition Examination Survey (KNHANES), 915 cancer survivors and 29,694 controls without a history of cancer were selected. Serum 25(OH)D levels were measured; vitamin D deficiency was defined as 25(OH)D levels less than 20 ng/mL. Chi-square tests and multiple logistic regression analyses were used to evaluate the prevalence of vitamin D deficiency and associated factors.

**Results:**

Vitamin D deficiency was observed in 62.7% of cancer survivors and 67.1% of controls. Among cancer survivors, vitamin D deficiency was most prevalent among 19–44 year olds (76.2%) and among managers, professionals, and related workers (79.3%). Multiple logistic regression analysis revealed that younger cancer survivors and those who work indoors were predisposed to vitamin D deficiency.

**Conclusion:**

Vitamin D deficiency was prevalent among both cancer survivors and controls in Korea. The regular evaluation and management of vitamin D levels is needed for both bone health and general health in cancer survivors.

## Introduction

As people spend more time indoors at home or in an office environment, they fail to obtain enough sunlight to adequately produce cutaneous vitamin D [1]. Thus, vitamin D deficiency has become a major health problem in modern society. Recent studies have consistently reported a surprisingly high prevalence of vitamin D deficiency across all age groups worldwide [2–4].

The worldwide prevalence of vitamin D deficiency is 30–50% among the general population [2, 5]. Vitamin D deficiency is common in Korea. In a study using data from the Korea National Health and Nutrition Examination Survey (KNHANES), the mean serum level of 25(OH)D was 21.2±7.5 ng/ml in males and 18.2±7.1 ng/ml in females. Vitamin D deficiency (which is defined as less than 20 ng/ml) was found in 47.3% of males and 64.5% of females; only 13.2% of males and 6.7% of females had a serum 25(OH)D level of greater than 30 ng/ml [3].

Vitamin D plays an important role in bone and mineral metabolism, and its deficiency is associated with the development of metabolic bone diseases such as rickets and osteomalacia [6]. Recently, several health conditions have been associated with the non-skeletal actions of vitamin D, including cardiovascular diseases, diabetes mellitus, infection, and autoimmune diseases [5, 7, 8].

Several epidemiological studies have reported an association between vitamin D deficiency and cancer. Higher vitamin D levels have been associated with decreased mortality from breast [9, 10], colon [10, 11], and liver cancers [12]. In addition, vitamin D from dietary intake and sunlight exposure has been associated with a reduced risk of cancer [13, 14].

There are limited reports that have examined the prevalence of vitamin D deficiency among cancer survivors. In 2007, a U.S. study in 99 breast cancer survivors and 54 controls reported that vitamin D deficiency (<32 ng/mL) was observed in 76 of 99 (77%) of the breast cancer survivors and 51 of 54 (94%) of the controls [15]. In a different multiethnic cohort study in the U.S., the mean vitamin D level was 24.8±10.4 ng/mL, and 75.6% of the 790 breast cancer survivors had low serum 25(OH)D [16]. Another study with a large, diverse population of cancer survivors who were followed in a childhood cancer survivor clinic reported a mean vitamin D level of 25.2±10.37 ng/ml, and 29% of the patients were 25(OH)D insufficient (<20 ng/ml) [17].

Vitamin D levels and the prevalence of vitamin D deficiency have not been adequately determined among cancer survivors in Korea. Therefore, we investigated the vitamin D levels and the prevalence of vitamin D deficiency among cancer survivors in Korea. We also investigated the difference between cancer survivors and controls without a history of cancer, and identified the predictors for vitamin D deficiency among cancer survivors.

## Materials and Methods

### Data source

This study is based on the 4th (2007–2009) and 5th (2010–2012) Korea National Health and Nutrition Examination Survey (KNHANES). The KNHANES was a cross-sectional and nationally representative survey conducted by the Korea Centers for Disease Control and Prevention (KCDC). A rolling sampling method with a stratified, multistage probability sampling design was used to assess the health status of the non-institutionalized civilian population in Korea. The survey collected data via household interviews and by direct standardized physical examinations conducted in specially equipped mobile examination centers. Detailed information on the design of the survey has been provided elsewhere [18].

Serum 25(OH)D levels were obtained starting in the second year (2008) of KNHANES IV. The KNHANES response rates were 77.8% (9,744 of 12,528) in 2008, 82.8% (10,533 of 12,722) in 2009, 81.9% (8,958 of 10,938) in 2010, 80.4% (8,518 of 10,589) in 2011, and 80.0% (8,058 of 10,069) in 2012, respectively. Among a total of 45,811 participants, 11,141 participants were under 19 years old and 4,061 participants were missing serum 25(OD)D level data; these participants were excluded. A total of 30,609 adults with a serum 25(OH)D level were included in this study. All survey protocols were approved by the Institutional Review Board of the KCDC, and all participants provided informed consent.

### Cancer survivors

Cancer survivors were defined as those who answered "yes" to the question, ‘‘Have you ever been told by a doctor that you had cancer?”. Cancer survivors were asked about the site of their cancer and the age at diagnosis. The time since diagnosis was calculated as the difference between the age at the time of the survey and the age at diagnosis. Controls were defined as subjects who reported no cancer history.

### Vitamin D levels

Blood samples were processed and refrigerated. The samples were transported on the day of the survey to the designated central laboratory of Neodin Medical Institute (NMI), a laboratory certified by the Korean Ministry of Health and Welfare in Seoul, Korea. Blood samples were analyzed within 24 hours after transport. Serum 25(OH)D levels were measured by radioimmunoassay methods with a WIZARD 1470 Gamma Counter (PerkinElmer, Finland) and a Gamma Counter (Hewlett Packard, USA) in the 4th and 5th KNHANES, respectively [19]. Vitamin D deficiency was defined as a level of <20 ng/ml as per the recent guidelines proposed by the Institute of Medicine (IOM).

### Covariates

Several covariates such as sex, age, occupation type, residence area (urban, rural), regular walking, physical activity, body mass index (underweight [<18.5 kg/m^2^], normal [18.5–24.9 kg/m^2^], or obese [≥25 kg/m^2^],), chronic condition, and survey year also were collected. Occupation was classified into seven groups according to the standard Korean classification of occupation: 1) managers, professionals, and related workers; 2) clerks; 3) service and sales workers; 4) skilled agricultural, forestry, and fishery workers; 5) craft, equipment, machine operating, and assembling workers; 6) elementary workers; and 7) unemployed (e.g., housewife, student). Non-cancer co-morbid conditions included hypertension, diabetes mellitus, stroke, heart disease, asthma, pulmonary tuberculosis, hepatitis B, C, liver cirrhosis and renal failure.

### Statistical analysis

The general subject characteristics were reported as the number and percentage and the general characteristics by cancer history were compared with chi-square tests. The difference in the prevalence of vitamin D insufficiency between cancer survivors and controls was tested using Chi-square tests. Multiple logistic regression analyses were used to examine factors associated with vitamin D deficiency in cancer survivors and non-cancer controls. All statistical analyses were conducted using SAS V9.3 (SAS Institute, Cary, NC, USA). A p<0.05 was considered statistically significant.

## Results

### Characteristics of cancer survivors and controls

The general characteristics of cancer survivors and controls were significantly different. The cancer survivors were more likely to be women, elderly, and unemployed, and were more likely to have a non-cancer chronic disease. The most common diagnosis was stomach cancer (19.9%), and 49.1% of survivors were diagnosed more than 5 years before the survey was conducted (Table 1).

**Table 1:** Characteristics of cancer survivors and controls.

Characteristics	Self-reported history of cancer(n = 915)	No reported cancer history(n = 29694)	p-value
Sex			<0.001
Men	327(35.7)	12850(43.3)	
Women	588(64.3)	16844(56.7)	
Age (years)			<0.001
19–44	101(11.0)	12690(42.7)	
45–64	433(47.3)	10615(35.8)	
≥65	381(41.6)	6389(21.5)	
Occupation			<0.001
Managers, professionals, and related workers	58(6.3)	3597(12.1)	
Clerks	36(3.9)	2327(7.8)	
Service and sales workers	78(8.5)	3692(12.4)	
Skilled agricultural, forestry, and fishery workers	104(11.4)	2572(8.7)	
Craft, equipment, machine operating and assembling workers	38(4.2)	2812(9.5)	
Elementary workers	58(6.3)	2588(8.7)	
Unemployed (e.g., housewife, student)	543(59.3)	12106(40.8)	
Residence area			0.190
Urban	583(63.7)	19540(65.8)	
Rural	332(36.3)	10154(34.2)	
Regular walking			0.082
No	514(56.2)	17532(59.0)	
Yes	401(43.8)	12162(41.0)	
Physical activity			0.953
No	717(78.4)	23244(78.3)	
Yes	198(21.6)	6450(21.7)	
Body mass index			0.529
Underweight	47(5.2)	1335(4.5)	
Normal	588(64.4)	18875(63.8)	
Overweight	278(30.5)	9377(31.7)	
Co-morbid non-cancer conditions^a^			<0.001
No	491(53.7)	20873(70.3)	
Yes	424(46.3)	8821(29.7)	
Survey year			0.094
2008	160(17.5)	5820(19.6)	
2009	197(21.5)	6989(23.5)	
2010	176(19.2)	5766(19.4)	
2011	197(21.5)	5716(19.3)	
2012	185(20.2)	5403(18.2)	
Age at diagnosis (years)			
19–44	247(27.0)		
45–64	521(57.0)		
≥65	146(16.0)		
Time since diagnosis (years)			
<1	137(15.0)		
<5	328(35.9)		
≥5	448(49.1)		
Cancer site			
Stomach	182(19.9)		
Liver	21(2.3)		
Colorectum	83(9.1)		
Breast	119(13.0)		
Cervix	117(12.8)		
Lung	23(2.5)		
Thyroid	40(4.4)		
Other	330(36.1)		

Data are expressed as number (%).

^a^Co-morbid non-cancer conditions included hypertension, diabetes mellitus, stroke, heart disease, asthma, pulmonary tuberculosis, hepatitis B or C, liver cirrhosis, and renal failure.

### Vitamin D levels and the prevalence of vitamin D deficiency in cancer survivors and controls

The mean vitamin D level in cancer survivors was 18.6±18.1 ng/ml, which was significantly higher than the mean level of 18.1±18.0 ng/ml among controls (p = 0.029).

Vitamin D deficiency (<20 ng/ml) was found in 62.7% of cancer survivors and 67.1% of controls (p = 0.006). The multiple logistic regression analysis revealed that the adjusted odds ratio (aOR) for vitamin D deficiency was similar among cancer survivors and non-cancer controls after controlling for several covariates (aOR = 0.96, 95% confidence interval = 0.83–1.12).

### Factors associated with vitamin D deficiency in cancer survivors and controls

The prevalence of vitamin D deficiency was highest among survivors aged 19–44 (76.2%) and managers, professionals, and related workers (79.3%). These figures were similar to those obtained in non-cancer controls by age group and occupation type (Table 2).

**Table 2:** Factors associated with vitamin D deficiency in cancer survivors and controls.

	Self-reported history of cancer		No reported cancer history	
	% of vit Ddeficiency	p-value	% of vit Ddeficiency	p-value
Total	62.7		67.1	
Sex		0.382		<0.001
Men	60.9		58.8	
Women	63.8		73.4	
Age (years)		0.005		<0.001
19–44	76.2		76.2	
45–64	63.0		62.6	
≥65	58.8		56.6	
Occupation		<0.001		<0.001
Managers, professionals, and related workers	79.3		74.8	
Clerks	77.8		74.7	
Service and sales workers	61.5		72.8	
Skilled agricultural, forestry, and fishery workers	39.4		38.5	
Craft, equipment, machine operating, and assembling workers	57.9		61.6	
Elementary workers	60.3		62.6	
Unemployed (e.g., housewife, student)	65.2		69.9	
Residence area		0.002		<0.001
Urban	66.6		71.8	
Rural	56.0		58.1	
Regular walking		0.146		<0.001
No	64.8		69.1	
Yes	60.1		64.3	
Physical activity		0.489		<0.001
No	63.3		68.7	
Yes	60.6		61.2	
Body mass index		0.873		<0.001
Underweight	63.8		73.3	
Normal	63.3		67.4	
Overweight	61.5		65.7	
Co-morbid non-cancer conditions^a^		0.782		<0.001
No	62.3		69.5	
Yes	63.2		61.5	
Survey year		0.574		<0.001
2008	58.8		56.2	
2009	61.9		66.1	
2010	61.4		67.9	
2011	67.0		71.3	
2012	63.8		74.8	
Time since diagnosis (years)		0.292		
<1	68.6			
<5	61.0			
≥5	62.5			
Cancer type		0.595		
Stomach	61.5			
Liver	81.0			
Colorectum	61.4			
Breast	64.7			
Cervix	66.7			
Lung	65.2			
Thyroid	55.0			
Other	61.2			

The multiple logistic regression analysis revealed that the younger age group had a significantly higher odds ratio for vitamin D deficiency in both groups after controlling for several covariates. In addition, several occupation types (managers, professionals, and related workers; clerks; service and sales workers) were more likely to have vitamin D deficiency compared with agricultural, forestry, and fishery workers in both the cancer survivor and non-cancer control groups. The factors associated with cancer, such as cancer type and cancer duration, were not associated with vitamin D deficiency. Among the controls, individuals who resided in an urban area, did not report regular walking or physical exercise, women, and respondents from the most recent survey year were associated with higher odds for vitamin D deficiency, but these factors were not associated with vitamin D deficiency among cancer survivors (Table 3).

**Table 3:** Adjusted odds ratios (95% confidence interval) for vitamin D insufficiency in cancer survivors and controls.

	Self-reported history of cancer	No reported cancer history
Sex(/men)		
Women	1.07(0.74–1.54)	1.97(1.87–2.08)
Age (/≥65)		
19–44	1.97(1.06–3.67)	2.09(1.93–2.27)
45–64	1.19(0.85–1.66)	1.20(1.12–1.29)
Occupation(/skilled agricultural, forestry, and fishery workers)		
Managers, professionals, and related workers	4.19(1.86–9.42)	2.68(2.37–3.03)
Clerks	3.68(1.44–9.41)	2.55(2.23–2.91)
Service and sales workers	2.05(1.05–4.02)	2.51(2.23–2.82)
Craft, equipment, machine operating, and assembling workers	1.48(0.63–3.46)	2.05(1.82–2.32)
Elementary workers	1.93(0.97–3.85)	1.89(1.68–2.13)
Unemployed (e.g., housewife, student)	2.40(1.48–3.88)	2.18(1.98–2.40)
Residence area(/rural)		
Urban	1.28(0.94–1.75)	1.45(1.38–1.54)
Regular walking (/yes)		
No	1.21(0.90–1.64)	1.11(1.05–1.17)
Physical activity (/yes)		
No	1.03(0.73–1.47)	1.19(1.12–1.26)
Body mass index (/overweight)		
Underweight	1.01(0.50–2.05)	1.12(0.97–1.28)
Normal	1.09(0.78–1.51)	1.00(0.95–1.06)
Co-morbid non-cancer conditions^a^ (/no)		
Yes	1.15(0.84–1.56)	1.04(0.98–1.11)
Survey year (/2008)		
2009	1.02(0.65–1.60)	1.57(1.45–1.69)
2010	0.95(0.59–1.53)	1.66(1.53–1.79)
2011	1.26(0.79–2.01)	2.00(1.85–2.17)
2012	1.16(0.72–1.87)	2.39(2.20–2.61)
Time since diagnosis (/≥5 years)		
<1	1.19(0.77–1.86)	
<5	0.86(0.62–1.19)	
Cancer type (/stomach)		
Liver	2.30(0.70–7.52)	
Colorectum	0.84(0.46–1.51)	
Breast	0.82(0.47–1.45)	
Cervix	1.01(0.57–1.78)	
Lung	1.07(0.39–2.92)	
Thyroid	0.57(0.26–1.26)	
Other	0.78(0.51–1.19)	

## Discussion

This study investigated the prevalence of vitamin D deficiency and examined the factors associated with vitamin D deficiency among cancer survivors in Korea. The prevalence and associated factors also were compared with non-cancer controls. Approximately 62.7% and 67.1% of cancer survivors and controls, respectively, exhibited vitamin D deficiency. Age and occupation type were associated with vitamin D deficiency among cancer survivors.

Because many cancer survivors can expect a long survival time post-diagnosis, maintaining quality of life following treatment is an important goal. The assessment of vitamin D deficiency should be an important strategy in cancer survivors. Cancer patients are acutely at risk for osteoporosis due to various cancer treatments, including hormonal therapy, chemotherapy, and glucocorticoids, which increase bone resorption and decrease bone marrow density [20]. Vitamin D also affects cancer prognosis through its effects on cellular proliferation, differentiation, apoptosis, and angiogenesis [21].

This study found that more than half of cancer survivors (62.7%) exhibited a vitamin D deficiency. High rates of vitamin D deficiency among cancer survivors in Korea are consistent with previous results from other countries. The prevalence of vitamin D deficiency among breast cancer survivors ranged from 75.6% to 86% [15]. A recent review of vitamin D levels among cancer survivors revealed that the prevalence of vitamin D deficiency was more than 70% in many studies. The prevalence of vitamin D deficiency in cancer survivors varied by study population, sample size, study design, definition of vitamin D deficiency used, and method of vitamin D assessment [22]. However, most previous studies were limited to one particular type of cancer with relatively small sample sizes; therefore, the findings regarding the prevalence of vitamin D deficiency in these studies should be interpreted with caution.

The prevalence of vitamin D deficiency among cancer survivors was not significantly different from non-cancer controls. This result was inconsistent with previous studies. A previous cross-sectional study of 61 children with a history of malignancy revealed that vitamin D deficiency (<10 ng/ml) was more common among children with a malignant disease compared with controls (21.3% vs. 3.3%, respectively) [23]. However, a study among patients who completed breast cancer treatment revealed that most breast cancer survivors (77%) and control women (94%) had a vitamin D deficiency (<32 ng/mL). Furthermore, control women had significantly lower concentrations of vitamin D than breast cancer survivors [15]. In our study, the prevalence of vitamin D deficiency was markedly high in both cancer survivors and controls. These results might reflect a decrease in outdoor exposure due to lifestyle changes among the general Korean population rather than a result of the cancer itself.

The risk factors for vitamin D deficiency in our population all have been identified as risk factors for vitamin D deficiency in the general population. Only age and occupation type were found to affect the prevalence of vitamin D deficiency in cancer survivors. Cancer-specific variables such as the time since diagnosis and cancer site were not associated with a risk of vitamin D deficiency.

The cutaneous synthesis of vitamin D was decreased as age increased. Therefore, old age is considered a risk factor for vitamin D deficiency. The elderly may be at an additional risk of deficiency due to decreased mobility, which consequently leads to decreased sun exposure [24]. However, in our study, vitamin D deficiency was most prevalent among 19–44 years old and lowest among individuals 65 years and older. Recently, the higher prevalence of vitamin D deficiency among younger individuals was reported from several worldwide population-based data sets, including Korea. Vitamin D deficiency was most prevalent among 20–29 years old, with a rate of 65.0% among males and 79.9% among females in the 2008 KNHANES [3]. The prevalence of low vitamin D levels in the National Diet and Nutrition Survey of the U.K. during 1992–2001 averaged between 5% and 20% in most age groups, but was in the range of 20%–40% in young men and women (age range, 19–24 years) [25]. In addition, a study based on NHANES III (1988–2004) in the U.S. also revealed that serum 25(OH)D levels were lowest for those aged 20 to 39 years among some subgroup [26]. Although the factors associated with a greater risk of vitamin D deficiency among young adults are not clear, these factors may include behavioral factors such as an indoor lifestyle (e.g., the use of televisions, computers, and video games) and less time outdoors among younger compared with older individuals [26, 27].

The prevalence of vitamin D deficiency also was significantly different across occupation types. The prevalence of vitamin D deficiency was highest in managers, professionals, and related workers compared with skilled agricultural, forestry, and fishery workers. These results are consistent with previous studies in Korea [3, 28] and other countries [29–31]. This result may be because subjects who are classified as managers, professionals, and related workers are usually indoors and do not receive adequate sunlight exposure. Meanwhile, individuals engaged in skilled agriculture, forestry, and fishery often spend most of their time outdoors and have relatively higher serum levels of vitamin D.

In cancer survivors, the difference of the vitamin D deficiency between men and women did not exist. But, the vitamin D deficiency in women was significantly higher compared to that in men in non-cancer controls. Korean women were reported to have the lowest vitamin D level of all countries in a recent international study of 1,285 osteoporosis patients from 18 countries. Korean women preferred "fair skin", and they deliberately avoided sunlight and used sunscreen like other East Asian women [32].

This study has several limitations due to the nature of KNHANES. First, KNHANES did not collect data on the amount of sunlight exposure and other factors associated with the cutaneous synthesis of vitamin D such as sunscreen use, clothing, or time spent outdoors. We used other variables, such as occupation type and residence area, as a proxy for these variables. Although we could not estimate how the level of sunlight exposure actually differs among the various occupations and residence area, we assumed that subjects who work outdoors (e.g., agriculture, forestry, or fishery) or live in a rural area would have more sunlight exposure than subjects who work indoors or live in an urban area. Second, data about the dietary intake of vitamin D or the use of vitamin D supplementation was not collected. Finally, because the KNHANES only included non-institutionalized individuals, cancer survivors who were admitted to the hospital or in convalescence were not included in the study. Therefore, cancer survivors in this study may not be representative of the general cancer survivors in Korea.

The prevalence of vitamin D deficiency was high in both cancer survivors and controls in Korea. Vitamin D deficiency was associated with age group and occupation type in both controls and survivors. Because vitamin D is crucial to bone health, assessing vitamin D status and correcting for vitamin D deficiency should be a critical goal for both cancer survivors and the general population. Cancer survivors in particular are at a greater risk for subsequent malignancies, cardiovascular disease, and infection, and these risks might be associated with vitamin D levels. Therefore, our results suggest that improvements in vitamin D levels are needed among cancer survivors.
